# DNA End Joining: G0-ing to the Core

**DOI:** 10.3390/biom11101487

**Published:** 2021-10-09

**Authors:** Richard L. Frock, Cheyenne Sadeghi, Jodie Meng, Jing L. Wang

**Affiliations:** Division of Radiation and Cancer Biology, Department of Radiation Oncology, Stanford University School of Medicine, Stanford, CA 94305, USA; csadeghi@stanford.edu (C.S.); jodieme@stanford.edu (J.M.); jlwang90@stanford.edu (J.L.W.)

**Keywords:** NHEJ, A-EJ, DSB repair, non-cycling, end joining, synapse, tethering, V(D)J recombination, translocation, functional redundancy

## Abstract

Humans have evolved a series of DNA double-strand break (DSB) repair pathways to efficiently and accurately rejoin nascently formed pairs of double-stranded DNA ends (DSEs). In G0/G1-phase cells, non-homologous end joining (NHEJ) and alternative end joining (A-EJ) operate to support covalent rejoining of DSEs. While NHEJ is predominantly utilized and collaborates extensively with the DNA damage response (DDR) to support pairing of DSEs, much less is known about A-EJ collaboration with DDR factors when NHEJ is absent. Non-cycling lymphocyte progenitor cells use NHEJ to complete V(D)J recombination of antigen receptor genes, initiated by the RAG1/2 endonuclease which holds its pair of targeted DSBs in a synapse until each specified pair of DSEs is handed off to the NHEJ DSB sensor complex, Ku. Similar to designer endonuclease DSBs, the absence of Ku allows for A-EJ to access RAG1/2 DSEs but with random pairing to complete their repair. Here, we describe recent insights into the major phases of DSB end joining, with an emphasis on synapsis and tethering mechanisms, and bring together new and old concepts of NHEJ vs. A-EJ and on RAG2-mediated repair pathway choice.

## 1. Introduction

Cells are tasked with constant surveillance and maintenance of genome integrity by various sources of DNA-damaging agents [1,2,3,4] that generate DNA lesions, expose DNA strands, and form double-stranded DNA ends (DSEs) [5,6,7] that are typically the result of a double-strand DNA break (DSB). Thus, multiple and simultaneous DSBs represent the greatest risk for catastrophic genome dysregulation due to the stochastic exchange of DSEs that occur between them to form translocations. Generally, the rejoining or recombining of a DSB can be considered in four distinct phases of engagement: (1) Initiation, which senses and secures DSEs; (2) Determination, which induces DNA damage response (DDR) signaling via post-translational modifications to support repair pathway engagement; (3) Intervention, which integrates steps necessary for ligation such as resecting DNA strands, synthesizing DNA, processing damaged ends, and/or aligning ends; and (4) Resolution, which ligates DNA strands and extricates remaining repair factors from DNA.

There are two primary DSB repair pathways. Homologous recombination (HR), found in all cellular life [8], is a high-fidelity, yet high-energy and time-consuming [9], repair process that requires a template sequence and is active in the S/G2 cell cycle phases [10]. In contrast, eukaryotes and some bacteria/archaea species [11] possess non-homologous DNA end joining (NHEJ), which is classically described as a relatively rapid repair pathway that ligates both strands of two DNA ends [12,13]. NHEJ is active throughout interphase [14,15] and is regarded as the repair pathway of choice in G1-phase cells [10] with reasonably high fidelity; the generation of small insertions and deletions is influenced by the type of overhang generated and the type of base damage incurred [4,16,17,18,19,20]. Between these two repair pathway extremes lie additional, less understood, DSB repair pathways (i.e., single-strand annealing—SSA; alternative end joining—A-EJ) [21,22] that may possess specialized functions in mammals and/or provide additional redundancy to ensure repair at the cost of decreased fidelity [18,23,24,25,26].

Recent studies have provided new insights into NHEJ and A-EJ that have implications for therapeutic applications involving DSB repair in quiescent and cycling cells [18,27,28,29,30,31,32,33,34,35,36]. In this review, we discuss DNA end-joining structures and mechanisms at different phases of engagement, with an emphasis on pre-replicative cell cycle states, evolutionary conservation, and repair pathway choice during V(D)J recombination.

## 2. NHEJ Overview

**Figure 1:**
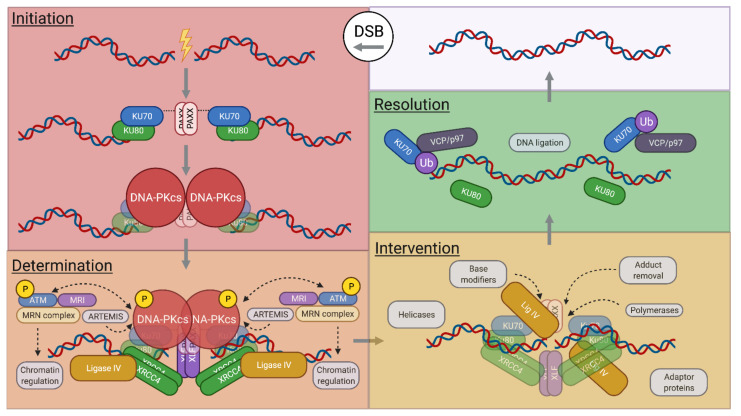
Four phases of DSB repair by NHEJ. Initiation: the NHEJ DSB sensing complex, Ku, recognizes and binds DSEs. PAXX dimers may keep DSEs paired by interaction with KU70. DNA-PKcs binds to Ku to form DNA-PK. Determination: DNA-PK is phosphorylated by ATM and vice versa; MRI mediates their interaction but no clear mechanism beyond binding. ATM and the MRN complex mediate chromatin remodeling of the neighboring chromatin to prime for repair pathway commitment. Phosphorylated DNA-PK activates ARTEMIS to open DNA hairpins and suppress DNA ligation during this process. Binding of XLF, XRCC4 and Ligase IV induces conformation changes to strengthen synapsis of paired DSEs. Intervention: hyper trans autophosphorylation induces release of DNA-PKcs and engagement for end compatibility using a toolkit of end-processing enzymes. Resolution: DSEs are ligated and constitutive damage signal dissolves as ubiquitinated Ku is removed from DNA by the ATPase VCP/p97.

As a “rapid response” DSB repair pathway [37], NHEJ can complete ligation of compatible broken ends in minutes [9]. However, incompatible ends require more time, needing to resect, modify, and/or synthesize nucleotides prior to ligation [4,38,39,40,41,42,43]. All NHEJ reactions require the KU70 and KU80 (“Ku”) DSE sensing complex and the XRCC4/Ligase IV ligation complex, which are considered core components to the NHEJ reaction (Figure 1). Their essentiality is best evidenced by evolutionary conservation down to yeast and in some bacterial and archaeal species [11,44], the greatest sensitivity to ionizing radiation (IR) when absent in cells, and their requisite role to complete V(D)J recombination [45,46,47,48,49,50,51,52]. In this regard, other NHEJ and associated factors may serve essential functions that are context specific or are functionally redundant with varying individual impacts to the overall efficiency of the reaction (Figure 1).

### 2.1. NHEJ Initiation

NHEJ senses DNA ends via the Ku complex [53]. The heterodimer is basket shaped, with each monomer encircling the DNA phosphate backbone [54]. Ku has high affinity for blunt ends [55] and a preference for the single-strand/double-strand (ss/dsDNA) interface in the context of ssDNA overhangs in vitro [56]. Ku loading onto ends will remove in its path damaged abasic or apurinic/apyrimidinic nucleotides from termini since it possesses 5′dRP/AP lyase activity [42]. Ku serves as the prime loading complex for recruiting other NHEJ factors to process ends, as needed, and to complete ligation between DNA ends. Ku alone contributes to DSE synapsis in bacteria but has no discernable contribution in humans [57], therefore, requiring additional factors for synapsis. In this regard, the DNA-dependent protein kinase (DNA-PK) catalytic subunit (DNA-PKcs), conserved in metazoans, interacts with the C-terminus of Ku80 [58,59] (thus, forming the DNA-PK DDR holoenzyme (Figure 1)), creates conformational changes in DNA-PKcs to stabilize DNA binding and enable synapsis of paired DSEs [27,28,30,31]. Functions of DNA-PKcs as a loading complex and as a synaptic mediator between DSEs are prompted by autophosphorylation and activation of the kinase activity of DNA-PKcs via twisting and stretching [31].

#### 2.1.1. NHEJ “Long-Range” Synapsis

**Figure 2:**
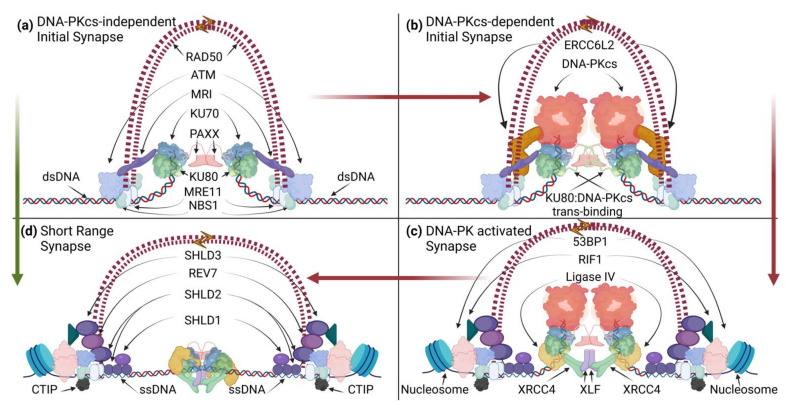
Model for dual-layer DSE pairing by NHEJ/DDR components in the G0/G1 phase. NHEJ proteins hold DSEs at the synapse while MRE11/RAD50 loops support DSE tethering a greater distance away from DNA ends. (**a**,**b**) Ku binds DSEs. PAXX dimerization may support synapsis across DSEs by KU70 interaction [62,63,64,65]. MRI connects KU70 to ATM signaling. Biophysical force transfer between synapsed ends, in response to Langevin Motion [82], signals constitutive twisting and stretching may activate ATM autophosphorylation and stabilize MRE11/RAD50 DNA binding through NBS1. (**b**) DNA-PKcs binds to Ku at end termini to form the DNA-PK holoenzyme. The C-terminal alpha helix of KU80 becomes bound to the opposite DNA-PKcs subunit to complete trans domain swapping and dimerization [27]. Stretching and twisting may prime for DNA-PK activation [31]. The presence of ERCC6L2 supports NHEJ functions, potentially in collaboration with MRI. (**c**) XLF dimer recruitment connects each DSE to flanking XRCC4 dimers and Ligase IV on each side [28,30]. The conformational shift reverts KU80 domain swapping, enabling ATM to activate DNA-PK and for DNA-PK to attenuate ATM activity to support NHEJ. NBS1/ATM chromatin signaling ultimately recruits 53BP1, RIF1, and the shieldin complex (SHLD1, SHLD2, REV7, and SHLD3) [83]. Biophysical detection of pairing may signal to commit to NHEJ. ATM phosphorylates CTIP (black, see (**d**)) to stimulate MRE11 nuclease activity. DNA-PK enables MRE11 endonuclease activity [32] to expose ssDNA for SHLD2 OB-fold domain binding (depicted here with 3′ strand nicking instead of 5′ strand nicking proposed for S/G2). (**d**) Complete DNA-PK trans autophosphorylation releases DNA-PKcs from the synapse, enabling transition to the short-range synapse [28,30]. Ligase IV becomes poised for ligation in a “close” synapse [36,84]. Note that NHEJ factors from (**a**) can readily proceed to the short-range configuration (green arrow; see (**d**)) in the absence of DNA-PKcs but may not be coordinated with MRN complex activities.

Synapsis of paired DSEs by DNA-PK, as first observed by single-molecule Förster resonance energy transfer (smFRET) studies [60], is configured in “long range” (defined as DSE pairs that are offset by >100Å) and may not require DNA-PK activation [36,60]. Recent cryo-EM suggest that synapsis of DNA-PK occurs by trans-swapping of Ku80 C-termini that interact with DNA-PKcs [27] (Figure 2) and may have additional functions beyond synapsis (e.g., priming DNA-PK activation by twisting and stretching of DNA-PKcs). A second “long-range” synapsis configuration determined by cryoEM [28,30], composed of DNA-PK with XRCC4-like factor (XLF) and XRCC4/Ligase IV is analogous to an earlier description of a closer synapse configuration than with DNA-PK alone [33,60,61]. Functionally, single-molecule DNA tension studies indicate that the “long-range” DNA-PK synapse is highly transient but can be further stabilized by Paralog of XLF and XRCC4 (PAXX) [62], which can interact directly with Ku70 [63,64,65]. Further, XLF can increase synapsis duration of DNA-PK-bound ends only with XRCC4/Ligase IV [62]. In this context, XLF supports synapsis as a dimer [61] and requires at least one Ku binding motif of the dimer and a sufficiently long C-terminus to support NHEJ resolution [66]. Thus, there appear to be two “long-range” synapse configurations, one that is transient and connected by DNA-PK, and another that is more stable that includes XLF and XRCC4/Ligase IV. Since DNA-PKcs is not required for all NHEJ, additional structures that include PAXX and other proposed tethering factors [67,68] may provide further insight into other possible early-stage synapse configurations (see model in Figure 2).

#### 2.1.2. NHEJ Filaments

XLF and XRCC4 are also phosphorylated by Ataxia-Telangiectasia Mutated (ATM) and/or DNA-PK DDR kinases [69,70], which decrease DNA interaction [71] and enable formation of alternating helical filaments [72,73,74,75,76]. These XLF:XRCC4 helical filaments reportedly form around DNA in cells, likely initiating from the DSB [77], and allow Ku with DSE to diffuse more readily within the confines of the filament [78], functioning to support synapsis and to aid in aligning ends. It is noteworthy that IFFO1, a nucleoskeleton protein that interacts with A-type lamin intermediate filaments, also interacts with XRCC4 in a putative scaffold role to immobilize DSEs and suppress translocations [79], perhaps to limit the three-dimensional search space and stabilize synapsis. However, while this activity with intermediate filaments is not expected to be universal in all vertebrate cells—nuclear A-type lamins are only expressed in more differentiated cells [80]—this variance in repair outcomes across normal somatic cells can also impact many cancers with aberrant lamin expression [81].

#### 2.1.3. NHEJ/DDR-Mediated Tethering

Sustained DSE pairing of a DSB is necessary to ensure their rejoining, and redundant mechanisms to keep DSEs together likely exist beyond their synapsis by NHEJ factors. The ATM DDR kinase is widely known for suppressing translocations and supporting V(D)J recombination [85,86]. How exactly ATM supports end-tethering functions is not fully understood but may be related to its activities at the DSB. ATM operates at the DSB via interaction of NBS1, which serves adapter functions for the multi-functional MRE11/RAD50/NBS1 (MRN) complex which can be recruited to DSBs under different contexts [87,88,89]. At the DSB, the Modulator of Retroviral Infection (MRI; aka CYREN) connects DNA-PK to ATM thorough its N-terminal Ku binding motif to KU70 and its C-terminal XLF-like motif (XLM) to ATM [90]. MRI is thought to communicate repair pathway choice by suppressing NHEJ in the S/G2 cell cycle phases [91], and its presence in quiescent B cell progenitors may further stabilize KU70:PAXX interactions [90]. This stabilization could be related to why PAXX plays a more significant NHEJ role in the G0/G1 phase [92,93]. ERCC6L2, identified in recent genetic screens [94,95,96], is a helicase that is also recruited to DSBs by NBS1 [97] and interacts with DNA-PKcs [98] and MRI [95] where it impacts repair pathway choice in cycling cells [94]. In terms of evolutionary conservation, MRE11 and RAD50 are found in virtually all organisms, while ATM, NBS1, MRI, ERCC6L2, PAXX, DNA-PKcs, and XLF are conserved in most if not all (i.e., ATM, NBS1, XLF) eukaryotes [8,44,63,99,100,101]. Thus, MRE11/RAD50 (MR) must provide core functions to support all DSB repair [37]. How the above-described factors then regulate or coordinate with MR-related activities is not entirely clear.

MRE11 is a structure-specific nuclease that senses DNA ends and typically functions with RAD50, an ATPase with long coiled-coil domains and zinc hook that can slide on DNA and bypass nucleosomes [102]. As a heterotetramer, the MR complex can tether with other MR complexes using cohesin-/condensin-like open and closed loop configurations [103,104,105]. Thus, models have been proposed suggesting that putative DSE tethering functions through interlinked RAD50 molecules [103,104,106,107,108]. The significance of many MRN-associated interactions has been laid out genetically as combined deficiency, but not any single deficiency, of either ATM, MRI, DNA-PKcs, ERCC6L2, or PAXX with XLF significantly inhibiting NHEJ; where assayed, this NHEJ inhibition is comparable to that of core NHEJ deficiencies with respect to IR sensitivity and block in V(D)J recombination [87,89,92,93,109,110,111,112,113]. Therefore, both the role of XLF to support NHEJ synapsis and the putative role of the MR complex to support tethering by a mechanism that involves ATM/DNA-PK signaling suggest that their functional redundancy would include proximal synapsis and distal tethering mechanisms to stabilize DSE pairing by supporting DSB rejoining and suppressing translocations (Figure 2) and warrants further investigation.

### 2.2. NHEJ Determination

DNA damage signaling to flanking chromatin is coordinated by ATM and DNA-PK, with ATM responsible for signal amplification using chromatin loop extrusion and diffusion [114,115]; reviews dedicated to chromatin remodeling and DSB movements in response to DNA damage are found here [116,117,118]. Ku and recruited NHEJ factors occupy DNA ends and can recruit end-processing enzymes as part of generating compatible ends for ligation [18,39,119] while also suppressing resection enzymes that promote homology-mediated repair pathways. Therefore, extraction of Ku from DSEs is necessary to complete repair by other pathways. Although the determination to switch from NHEJ to other repair pathways is minimal in G0/G1-phase cells compared to in the S/G2 phases, factors involved in this transition may be relevant for complex end processing with NHEJ or for supporting A-EJ. MRE11 is widely considered the nuclease to execute changes to DNA end access and to contribute to short-range resection. Normally, MRE11 cannot bind to DNA when interacting with ATP-bound RAD50 [120,121,122,123]. However, MRE11 3′ to 5′ exonuclease activity [124,125] is stimulated by the phosphorylated C-terminal binding protein (CtBP)-interacting protein (CTIP) when recruited by NBS1 [126,127,128]. MRE11 also possesses endonuclease activity for either DNA strand in this active state but only near DNA ends that are blocked by bound protein (e.g., DNA-PK) [129] or with protein-DNA adducts in the S phase when MRN is paired with phosphorylated CTIP and BRCA1 [130]. However, MRE11 exonuclease activity in G1-phase human cells also requires phosphorylated CTIP and BRCA1 in collaboration with NHEJ to repair complex ends from IR-induced DSBs [39]. In this regard, the context of CTIP phosphorylation can have different impacts to the level of resection and whether MRE11 endonuclease activity is stimulated [32]. Multiple CTIP phosphorylation sites exist and are differentially targeted by S/G2 CDKs [131], Ataxia-Telangeictasia and Rad3 related (ATR) [132], ATM [133,134], Polo-like kinase 3 [38], and DNA-PK [32].

Aside from promoting resection by CTIP phosphorylation, ATM also suppresses resection by initiating multi-stage recruitment of 53BP1, RIF1 and the Shieldin complex (composed of SHLD3, REV7, SHLD2, and SHLD1), which connects nearby chromatin to resected DNA near DSEs [6,83]. Resection in quiescent human and mouse cells are additionally regulated independently of 53BP1 by the dimerization partner, RB-like, E2F and multi-vulval class B (DREAM) complex components. Specifically, LIN37 cooperates with pRB, p107, and p130 to promote quiescence [135] and was identified in a genetic screen that induced resection of IR-generated DSBs in Ligase IV-deficient non-cycling progenitor B cells [29]. LIN37 functions to repress protein expression of key HR genes (i.e., BRCA1/2, BARD1, RAD51, BLM, and FANCD2) that can initiate resection in the G0 phase in its absence [29]. These striking findings suggest a clear differentiation of available DSB repair components between cycling G1 phase vs. quiescence and imply other non-cycling cell states [136,137,138] may also possess such differences.

### 2.3. NHEJ Intervention

DNA-PK can phosphorylate ATM to suppress its activity and promote NHEJ [139]. DNA-PK can also be activated via ATM trans-phosphorylation to suppress ligation [140], allowing for the recruitment of the ARTEMIS endonuclease by Ku [141]. ARTEMIS, activated by DNA-PK, opens DNA hairpins [142] and processes other DNA end structures [143] to form 3′ overhangs [16,143]. DNA-PK autophosphorylation associated with hairpin-sealed ends is stepwise, with full autophosphorylation present once the hairpin is opened [144]. Complete DNA-PK trans autophosphorylation in the “long-range” synapse variant with XLF and XRCC4/Ligase IV uncouples DNA-PKcs from the synapse to form a “short-range” synapse [28,30] that facilitates, as needed, processing enzymes, polymerases and tethering factors (i.e., TDP1, PNKP, APTX, POL µ, POL λ, TDT, APLF, WRN) [12,67,68,145] to support end alignment and ligation. This is consistent with mouse studies of kinase dead DNA-PKcs linking trans autophosphorylation with completion of hairpin-ended NHEJ [140,146] and provides insight into the DNA-PKcs-independent mechanisms of NHEJ. In this latter context, DSEs that are held in a transient “flexible” lateral-ended synapse by core NHEJ factors become aligned end to end as a stable “close” synapse in the presence of either PAXX or XLF [36,84] and may represent, in part, precursor or variant conformations of the “short-range” synapse [77].

### 2.4. NHEJ Resolution

Aligned and compatible ends are then ligated by Ligase IV. Although the trigger to initiate post-ligation dissociation is not known, extraction of Ku rings from repaired DNA is the crucial linchpin for NHEJ dissociation. KU70 phosphorylation destabilizes end affinity in association with the transition to HR [147] but does not appear to be important for extraction from intact DNA. Instead, K48-linked polyubiquitination on multiple sites of KU80, and in some cases KU70 (by RNF126 [148], RNF8 [149,150,151] or NEDD8-dependent [152] E3 ligases)—RNF138 does this only in the S/G2 phases [153,154]—leads to extraction from chromatin [148] by the VCP/p97 AAA+ ATPase [155] (Figure 1). In this regard, chromatin-modified DNA damage signals remain despite NHEJ ligation until Ku is extracted by p97 [155], clearly demonstrating that Ku extraction must first occur before chromatin damage signals are reverted. Uncoupling MRN activation on newly repaired DNA also requires ubiquitination of MRE11 [156] by VCP/p97 [157].

A speculative G0-phase NHEJ and DDR support model that integrates recent findings would include the following steps: (1) Ku or DNA-PK synapsis that includes PAXX and support from MRI, ATM, ERCC6L2, and the MRN complex, primarily functioning to keep ends synapsed until XLF and XRCC4/Ligase IV are recruited to form the more stable “long-range” (with DNA-PK) or “short-range” synapse (with Ku) [27,28,30,31]. Hyperphosphorylation of XLF and XRCC4 stabilizes filaments around Ku-bound DSEs may support synapsis [71], and MRN may coordinate with the Shieldin complex to limit resection and support synapsis. (2) Activated ARTEMIS would operate in a “long-range” synapse that includes XLF and XRCC4/Ligase IV [144], with accessory NHEJ factors acting on ends in “short range” as a laterally arrayed “flexible” synapse [84] after DNA-PKcs leaves the complex. (3) The various processing intermediates then structurally converge to a common “close” synapse for ligation by Ligase IV (Figure 2).

## 3. A-EJ Overview

A-EJ can be defined as end joining that occurs in the absence of core NHEJ factors, and many studies have implicated participating factors [15,18,24,34,158,159,160,161,162,163,164,165,166,167,168,169,170,171,172,173,174,175,176,177,178,179,180,181,182,183,184,185,186]. Most notable in recent years is polymerase theta (POL θ) [163,170,182], a multipurpose polymerase with RNA-templated DNA repair capabilities [187] that is upregulated in cancers [24,167,188]. Interpreting A-EJ contributions in the S/G2 cell cycle phases can be difficult since there are specialized repair processes and repair pathway/component redundancies that are still awaiting full elucidation [161,171,175,184,189,190]. Unlike NHEJ factors, which are mostly exclusive, many implicated A-EJ factors also participate in base excision repair (BER) and nucleotide excision repair (NER) [191], with commonly structured repair intermediate parallels that also extend to Okazaki fragment ligation during lagging strand DNA synthesis [192,193,194]. Although cells exposed to lesion-promoting DNA damage from IR and chemotherapeutics (e.g., interstrand crosslinks) would rely on both single- and double-strand DNA repair mechanisms, ascribing a physiological role to A-EJ has remained elusive due to the predominance and efficiency of NHEJ and HR. Thus, A-EJ may be regarded as inadvertent ultimate backup DSB repair pathways to prevent the persistence of DSEs [195].

### 3.1. A-EJ Pathways and Cell Cycle Dependence

While A-EJ is still not fully understood, recent studies with murine progenitor B cells identified separable A-EJ pathways [18,34]. In the G0 phase, the absence of Ku, reveals a bona fide A-EJ pathway separate from NHEJ factor engagement [18] and employs the XRCC1/DNA Ligase IIIα complex [172] since DNA Ligase I is not active in the G0 phase [18,196,197,198,199]; the alternative splice form that does not include XRCC1 binding, Ligase IIIβ, does not localize to the nucleus and is essential for mitochondrial DNA repair [172]. Correspondingly, A-EJ of targeted DSEs in the absence of the NHEJ ligation complex is blocked in the G0 phase. This demonstrates the level of NHEJ predominance over A-EJ [18,34] and explains why Ku deficiency can rescue the embryonic lethality of Ligase IV deficiency [200]. However, cells with unrepaired G0-phase DSBs, but then permitted to re-enter cycling phases and bypass cell cycle checkpoints [201,202], can use an NHEJ-variant A-EJ pathway (i.e., requiring extraction of Ku and NHEJ machinery from DSEs) that is dependent on POL θ [34]. Surprisingly, POL θ does not contribute to G0-phase A-EJ since it is not expressed during quiescence [34]. Thus, a working model for bona fide core A-EJ factors would include PARP1, MRE11, and XRCC1/Ligase IIIα.

### 3.2. G0-Phase A-EJ Initiation and Determination

**Figure 3:**
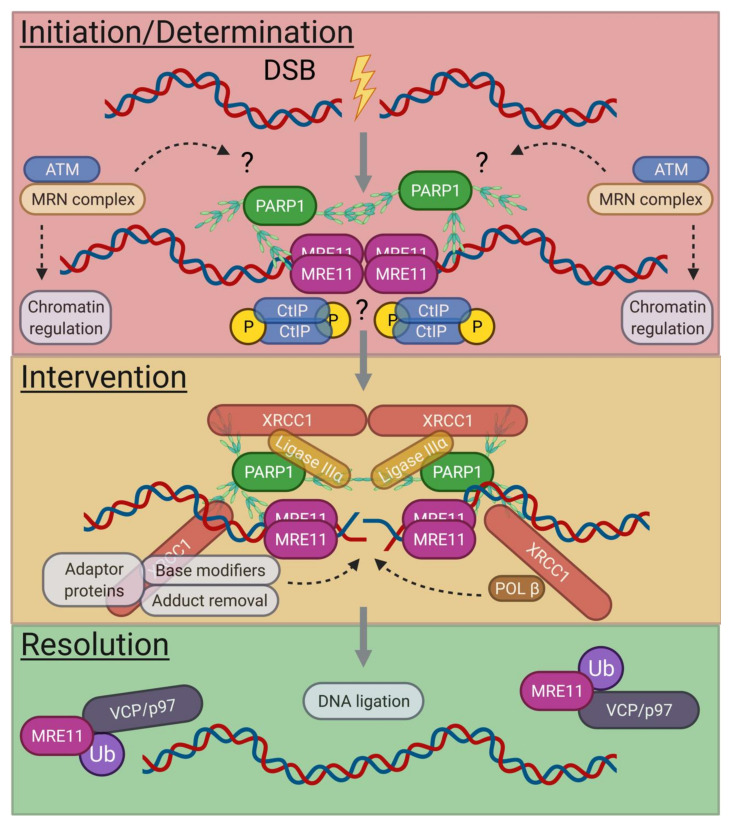
A speculative model for DSB repair by A-EJ in G0-phase cells. Quiescent B cell progenitors do not express POL θ and DNA Ligase I and instead use short-patch BER machinery (PARP1, XRCC1, Ligase IIIα, POL β, etc.) to complement MRE11 and other MRN-related DDR and tethering functions. Other factors (e.g., TREX1 and CTIP) may support A-EJ and require further investigation. After DNA ligation, MRE11 is ubiquitinated [157], allowing for removal of MRE11 and associated complexes from DNA and dissolution of DNA damage signals.

In non-cycling progenitor B cells, Ku suppresses A-EJ, rendering ends more susceptible to resection and A-EJ in its absence [18]. Similar to Ku, poly(ADP-ribose) polymerase 1 (PARP1) is a rapid responder to DSBs [174,203]. PARP1 senses and secures DNA nicks, ssDNA patches, and DSEs as part of its multiple roles in BER, NER, and A-EJ [204]. PARP1 is 10-fold more abundant than its functionally overlapping relative, PARP2 [12]. While no clear orthologs have been described, poly(ADP-ribosyl)ation is found in bacteria and archaea [205]. Individual gene deficiencies in mice are viable; however, combined deficiencies of PARP1/PARP2, PARP1/KU80, or PARP1/ATM are early-stage embryonic lethal [206,207,208], underscoring the significance of PARP1 in supporting DNA repair and collaborating with the DDR. PARP1 generates poly(ADP)ribose (PAR) adducts on proteins, can crosslink to DNA [209] and can recruit MRE11 and NBS1 to DSEs [88], where MRE11 may function to bridge ends rather than promote resection [88,103,210] (Figure 3). CTIP can promote A-EJ in the absence of Ku in cycling cells [185], multimerize into filaments [211,212] and support end hybridization [213]. As discussed earlier for NHEJ [103,104,106,107,108], the MR complex may also provide end-tethering functions for G0-phase A-EJ. However, without DNA-PK present, short-range resection occurs [18] and tethering functions may be affected by the ATR DDR kinase [132,214,215], which is recruited to the ss/dsDNA interface to sustain and coordinate repair even in the G1 phase [214,215]. In this context, it is not clear whether other resection enzymes or inhibitors function in the G0 phase [216,217,218,219,220,221].

### 3.3. G0-Phase A-EJ Intervention and Resolution

As with BER/NER, PARylation serves to recruit additional repair factors and support end tethering. XRCC1 recruitment and binding to PAR [222] can suppress excessive PARylation in the context of BER [223] and may similarly apply for G0-phase A-EJ. XRCC1 is a scaffold protein that brings in, most notably, polymerase beta (POL β) and DNA Ligase IIIα (Figure 3). DNA Ligase III is conserved only in vertebrates [172], whereas POL β belongs to the Pol X family of polymerases along with NHEJ co-factors POL λ, POL µ, and TDT that all arose from a single precursor gene in bacteria [224]. When phosphorylated by casein kinase 2 (CK2), XRCC1 also recruits a trio of factors: Polynucleotide kinase 3′ phosphatase (PNKP) to prepare ends for ligation/polymerization, Aprataxin (APTX) to remove adducts from failed ligation [40,204], and Aprataxin and PNK-like factor (APLF), which does not have a definitive role with XRCC1 yet [225]. Intriguingly, all three can function in NHEJ, where they also bind to CK2-phosphorylated XRCC4 [226,227].

Together, the above referenced factors are implicated in “simple” or short-patch BER that is typified with single-nucleotide replacement or small gap filling [40,228] and may operate in a similar manner at synapsed DSEs for A-EJ (Figure 3). While the exact stepwise mechanism remains to be determined, extrapolation of BER functions is plausible. To handle incompatible ends, a polymerase with proof-reading capability would suffice. Unfortunately, POL β lacks 3′ to 5′ proof reading, is error prone, and has a relatively short range of processivity (<7 bp) [228,229]. However, TREX1 is a 3′ to 5′ exonuclease [230] that can translocate to the nucleus under genotoxic stress and interact with PARP1 [231] to trim mispaired 3′ termini of DSEs, potentially generated by POL β. Thus, the mutagenic property of POL β coupled with the trans “proof reading” of TREX1 would address how incompatible ends are managed. Indeed, multiple attempts to ligate or modify nucleotide with adducts from failed ligation of mismatched base pairing may lead to blunting of ends. This testable model also supports the observed accumulation of unrepaired DSEs and increased utilization of direct joins for G0-phase A-EJ [18].

## 4. End-Joining Pathway Utilization during V(D)J Recombination

**Figure 4:**
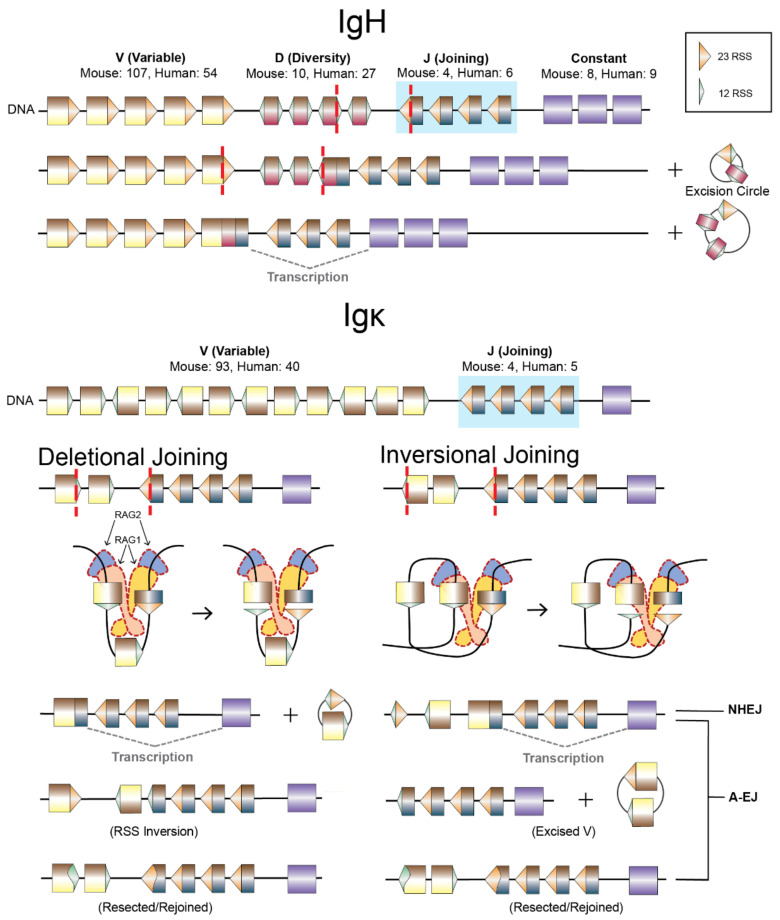
V(D)J recombination by NHEJ and V(D)J-like joining by A-EJ. The IgH and Igκ loci contain V, D, J and V, J gene segments that are used to generate the variable region of antigen receptors. Top: The IgH locus contains multiple constant region exons that are selected later in activated mature B cells during IgH class switch recombination to diversify antibody effector functions. Functional gene segments and exons for mouse and human Ig loci are enumerated from IMGT.org [237,238]. IgH undergoes D to J recombination first, followed by V to DJ recombination [239]; each recombination event generates excision circles of intervening DNA. In contrast, Igκ undergoes V to J recombination but V gene segments are arrayed in either a deletional or inversional orientation with respect to the J gene segments, resulting in deletions with excision circles (left) or retention of intervening sequence by inversion (right). V(D)J recombination begins with RAG1/2 (RAG) loading into recombination centers located in J gene segment clusters (blue box); the IgH RC also contains one D gene segment (not shown) [240]. RAG, with bound RC-associated RSS, directionally scans for 12/23 RSS pairing as DNA loops are extruded through CTCF/cohesin loop anchors [239]. RAG generates two DSBs to form two blunt RSS ends and two hairpin-sealed gene segment (coding) ends held in a PSC. NHEJ will proceed to open hairpins on coding ends, process, and join them, whereas RSS ends will be simply ligated, to complete V(D)J recombination. In the absence of the NHEJ DSB sensor, Ku, but not in the absence of the NHEJ ligation complex, A-EJ gains access to RAG DSEs, but pairing is random, resulting in V(D)J-like translocations, translocation of an RSS end to a different coding end, and resected/rejoined junctions associated with the initial RAG DSBs [18]. Thus, the rate of suitably paired antigen receptors (e.g., IgH:Igκ) would be very low and explains the leaky SCID phenotype of Ku-deficient mice [47].

Deficiency of core NHEJ factors blocks B and T lymphocyte development [45,46,47,51,201,232], resulting in a severe combined immunodeficiency (SCID). This defect is due to a failure to complete V(D)J recombination: a cut and paste reaction in the G0/G1 phase that brings together individual Variable (V) Diversity (D) and Joining (J) gene segments spread across several megabases in immunoglobulin (Ig) and T cell receptor (TCR) loci (Figure 4).

### 4.1. V(D)J Recombination

Recombination-Activating Gene (RAG) proteins (RAG1 and RAG2; “RAG”) are a domesticated transposase which initiate the cleavage event of V(D)J recombination but lost their ability to complete transposition [35,233,234]. RAG is heterotetramerically primed to seek a recombination sequence pair which, when recognized, provides the signal to initiate V(D)J recombination. Recombination Signal Sequences (RSSs) are composed of either a 12 bp or 23 bp non-conserved spacer sequence flanked on the incision side by a conserved heptameric “CAC/GTG” book-ended palindrome and an AT-rich nonamer on the opposite side. A 12/23RSS pairing by RAG1 is required and enforced by RAG2 [235] for RAG to coordinate a nick and subsequent double-strand cleavage at the interface between each RSS and their adjacent gene segment [35,236]. Each pair of DSEs formed are asymmetric with one blunt RSS end and one hairpin-sealed coding end, held in a post-cleavage synaptic complex (PSC). The RAG PSC collaborates with NHEJ to align the synapsis and eventual repair of coding ends, and separately RSS ends, that is characteristic of V(D)J recombination (Figure 4) [18].

The current model posits that RAG activation at recombination centers (RCs) [241] bind one RC-associated RSS with one RAG1/2 heterodimer and then proceeds to partner with a compatible RSS in the RC [240] or by scanning the appropriate DNA strand for a second RSS or potentially an off-target “CAC” motif [240,242,243] as chromatin loops are actively extruded [239,240,242,243,244,245,246,247]. However, recent single-molecule dynamics imaging suggests that RSS pairing in chromatin may be highly infrequent with low antigen receptor locus occupancy and transient residence times that would suggest sampling only a fraction of an extruded loop at any given time [248].

### 4.2. A-EJ of RAG DSEs, RAG2 Functions, and Repair Pathway Choice

A-EJ of RAG DSEs in quiescent B cell progenitors is handled similarly to endonuclease-targeted DSBs, with resection of ends and substantial levels of coding joins (mostly nonproductive) specifically for Ku deficiency [18], and not for XRCC4/Ligase IV deficiency [18,34]. These findings are also consistent with their respective leaky vs. complete SCID mouse phenotypes [45,47]. However, A-EJ of coding and RSS ends is not directed, as with NHEJ involvement, and instead results in unbiased joining of coding and RSS ends by a translocation-based mechanism that involves undirected end synapsis, likely after the PSC no longer sustains binding of all four ends (Figure 4) [18]. In this regard, deficiency in ATM or mutations in MRN can form hybrid joins (joining of an RSS end with another coding end) are formed in comparable frequency to coding joins when using a recombination reporter designed for inversional joins [133,249,250]. Hybrid joins would seemingly be the result of impaired MR complex DNA end tethers while in association with the RAG PSC and is consistent with an NHEJ functional redundancy that serves to maintain DSE pairing (Figure 2); whether such ends are repaired by aberrant NHEJ or by A-EJ remain to determined.

RAG is thought to shepherd its DSEs to NHEJ [134] rather than A-EJ since aberrant end joining and genome instability were detected by RAG2 C-terminal truncation mutations in the presence or absence of NHEJ [251,252,253,254]. Despite recent insights into G0/G1-phase A-EJ, it remains unclear how RAG2 C-terminal truncation promotes A-EJ. The RAG2 C-terminus, a partially acquired coincidence with a notable evolutionary stage of RAG domestication [35,234], restricts activity to the G0/G1 phase [255,256,257] and contains two domains: an acidic hinge and a plant homeodomain (PHD) zinc finger, that further modulate RAG activity. The acidic hinge is an intrinsically disordered domain, with negatively charged residues that stabilize the PSC [258] and additionally autoinhibits RAG activity [259,260]. The PHD finger binds histone H3K4me3 marks that are characteristic of transcription start sites and RCs located in J gene segment clusters of Ig and T cell receptor genes [261,262]; binding H3K4me3 marks disables its autoinhibition [259,260]. Thus, while the absence of the RAG2 C-terminus regulatory components is compatible with V(D)J recombination in mice, albeit with significantly impaired efficiency [263], the aberrant RAG-induced DSEs in S/G2 cell cycle phases may be subject to repair beyond NHEJ. Whether this repair involves end-joining pathways, is a result of restored DNA transposition [35], or a combination of both possibilities remains to be determined.

## 5. Conclusions

Many new insights into DNA end-joining mechanisms have come to light in recent months. Multi-subunit structures and single-molecule studies of NHEJ and DDR complexes have helped to piece together more of the rules of how the DSB repair machinery engages DSEs to synergize with genetic interpretations of repair factor deficiencies. Likewise, the determination of multiple A-EJ pathways, impact of A-EJ to V(D)J recombination, and differential regulation of HR gene expression will necessitate a re-evaluation of prior studies that applied G1- vs. G0-phase approaches to measure repair outcomes that should additionally encapsulate DNA excision repair pathways. More broadly, leveraging recent insights to identify synthetic genes for mitotically dormant cancer cells that contribute to recurrence and metastasis would be complementary to many existing therapies. They also highlight novel genome editing approaches for non-cycling cells, an important feature that most of our somatic cells share. However, central to our understanding of DNA end-joining pathways and toward developing effective therapeutics is exploring the nuanced differences in DNA repair between mice and humans [264,265,266,267,268,269,270].

## Data Availability

Not applicable.
